# “Lessons from Rare Forms of Osteoarthritis”

**DOI:** 10.1007/s00223-021-00896-3

**Published:** 2021-08-21

**Authors:** Rebecca F. Shepherd, Jemma G. Kerns, Lakshminarayan R. Ranganath, James A. Gallagher, Adam M. Taylor

**Affiliations:** 1 Lancaster Medical School, Faculty of Health & Medicine, Lancaster University, Lancaster, UK; 2 Departments of Clinical Biochemistry and Metabolic Medicine, Royal Liverpool University Hospital, Liverpool, L7 8XP UK; 3 Department of Musculoskeletal Biology, Institute of Ageing and Chronic Disease, University of Liverpool, Liverpool, L7 8T UK

## Abstract

Osteoarthritis (OA) is one of the most prevalent conditions in the world, particularly in the developed world with a significant increase in cases and their predicted impact as we move through the twenty-first century and this will be exacerbated by the covid pandemic. The degeneration of cartilage and bone as part of this condition is becoming better understood but there are still significant challenges in painting a complete picture to recognise all aspects of the condition and what treatment(s) are most appropriate in individual causes. OA encompasses many different types and this causes some of the challenges in fully understanding the condition. There have been examples through history where much has been learnt about common disease(s) from the study of rare or extreme phenotypes, particularly where Mendelian disorders are involved. The often early onset of symptoms combined with the rapid and aggressive pathogenesis of these diseases and their predictable outcomes give an often-under-explored resource. It is these “rarer forms of disease” that William Harvey referred to that offer novel insights into more common conditions through their more extreme presentations. In the case of OA, GWAS analyses demonstrate the multiple genes that are implicated in OA in the general population. In some of these rarer forms, single defective genes are responsible. The extreme phenotypes seen in conditions such as Camptodactyly Arthropathy-Coxa Vara-pericarditis Syndrome, Chondrodysplasias and Alkaptonuria all present potential opportunities for greater understanding of disease pathogenesis, novel therapeutic interventions and diagnostic imaging. This review examines some of the rarer presenting forms of OA and linked conditions, some of the novel discoveries made whilst studying them, and findings on imaging and treatment strategies.

## Introduction

Osteoarthritis (OA) is a disease of the joint that affects the cartilage and underlying bone. The term is used as an umbrella term to cover multiple pathologies that affect the articular musculoskeletal tissues [1].

OA is one of the most prevalent musculoskeletal conditions globally and it is on the increase as we move through the twenty-first century [2]. It has been suggested that there are factors such as increased life expectancy contributing to this rise, as populations live for longer thanks to the eradication of some diseases and improved standards of healthcare. Similarly, the ability of clinicians to detect signs of OA, particularly the earlier signs of the condition with increasing power and resolution of imaging techniques as well as the arrival of newer, more sensitive technologies has increased, potentially contributing to the increased numbers [3, 4]. There has been a worldwide increase in obesity and BMI levels in recent years, which may also contribute, in part, to the upwards reporting of OA [5–7]. The prevalence of OA and the associated disability has stimulated a global effort in OA research, but despite this global effort, there are currently no effective therapies. Furthermore, the pathogenesis of OA is not well understood. OA is considered to be multifactorial with both genetic and environmental factors contributing either solely or in combination. Genetic factors are becoming more evident and widely understood, but their associations with OA are usually joint specific, and in many cases are associated with other variables. For example, COMP and CHADL have been associated with a decreased age for hip replacement surgery for OA related disease [8]. Similarly, genomic associations for GDF-5, FTO and SMAD-3 have also been seen [9]. Genetic variants in ALDH1A2 which encodes retinaldehyde dehydrogenase 2 (RALDH2) have been identified as associated with the risk of developing hand OA [10] [11].

In the quest for understanding OA susceptibility and pathogenesis, GWAS studies have demonstrated the heterogeneity of genes implicated in the condition with hundreds of thousands of genomes being analysed (usually far more individuals than some of the rare diseases have global sufferers) to ascertain dozens of candidate genes in the susceptibility of OA [12]. This is further complicated by the fact that OA can affect different joints; hand, hip and knee being the most common and each anatomical site has its own genes that are linked to cause or pathogenesis, including but not limited to; hand including Wnt9A, TGFa, RUNX2, Col27 and GDF5 [13], hip including Col11A1, RUNX2, BMP5, SMAD3, IL11, GDF5 [14] and knee including DVWA, GDF5, Col11A1 and SMAD3 [15–17]. In this review, we put forward the proposition that more careful observation of extreme phenotypes could help elucidate the pathogenesis of OA and lead to new biomarkers and therapeutic targets. This idea is not new. The words of William Harvey, the great English physician of the 17th Century, are often cited *“Nature is nowhere accustomed more openly to display her secret mysteries than in cases where she shows tracings of her workings apart from the beaten paths; nor is there any better way to advance the proper practice of medicine than to give our minds to the discovery of the usual law of nature, by careful investigation of cases of rarer forms of disease”*. In extreme phenotypes of some Mendelian disorders, disease progression is often rapid and predictable; making it is easier to identify the initiation and advance of pathological changes. There are many examples from contemporary biomedicine of rare conditions helping to inform the development of new drugs. Research on familial hypercholesterolemia paved the way for the discovery of the blockbuster drugs statins, which are used to treat high cholesterol in millions across the globe [18, 19]. In bone, the observation that pyrophosphate was elevated in hypophosphatasia contributed to the development of bisphosphonates and more recently an increased understanding of a number of the rare high bone mass phenotypes, sclerosteosis and Van Buchem disease, has revealed the role of Wnt signalling and sclerostin in bone, leading to new drug targets and therapeutic interventions [20–24]. Inhibition of Cathepsin K, which is genetically deficient in the rare bone disease pycnodysostosis, is a target for anti-resorptive therapy.

In contrast to bone, studying rare diseases of cartilage is a relatively neglected area, but over the past few years some progress has been made in understanding rare cartilage syndromes. In this review we have selected some examples to highlight the potential of studying rare syndromes and hopefully to stimulate further research “apart from the beaten paths”.

### Camptodactyly Arthropathy-Coxa Vara-pericarditis Syndrome (CACP)

CACP (OMIM 208,250) is a rare autosomal recessive condition, first described in 1986, arising from genetic mutations in the gene coding for proteoglycan 4 (PRG4), with close to 100 mutation proven cases reported in the literature [25]. The widespread skeletal and non-skeletal manifestations include congenital camptodactyly, skeletal deformation including cox vara, early onset arthropathy, cataracts and pericarditis. Initial diagnosis is through clinical observation including X-rays and echocardiogram, with confirmation by synovial biopsy and/or genetic testing. Currently there are no specific therapies for CACP other than symptom relief. CACP shows symptom overlap with juvenile idiopathic arthritis (JIA), which can result in delay or initial mis-diagnosis, however medication used to treat JIA is not helpful for those with CACP [26].

The PRG4 gene, codes for a mucin- type proteoglycan, also known as lubricin, because of its key role as a lubricant at articular surfaces of joints and tendons. It is synthesised by chondrocytes at the articular surface and by synoviocytes and is present most abundantly in synovial fluid. However, the severe consequences of genetic deficiency of PRG4 indicate that it does more than act as a lubricant in joints, it is also present in the liver, lungs and heart [27].

Human synovial fluid from patients with CACP has shown a complete absence of C and N terminal peptides for lubricin, whereas it has been shown in synovial fluid from patients with both OA and RA. PRG4 deficient mice have been shown to exhibit synovial fluid protein deposition on cartilages, adhesion of synovial cells to the cartilage and show structural and biochemical alterations in the articular cartilage similar to osteoarthritic degeneration [28, 29]. Whilst there is no data on CACP models, the PGR4 therapies trialled in more routine OA models demonstrate positive effects [30]. Intra-articular injections of PRG4 in a rat and minipig models of OA have been shown to reduce cartilage damage [31–33] by restoring normal cartilage boundary lubrication function. Researchers are also trying to develop synthetic polymers which will be easier to produce and more resistant to break down [34, 35].

To avoid the requirement for multiple injections, Chen et al.have tested a gene therapy approach in surgical models of OA in mice in which PRG4 is over-expressed to promote chondroprotection. In these studies, interleukin-1 receptor antagonist (IL-1Ra) gene was also used to inhibit inflammation. PRG4 monotherapy maintained cartilage volume and covered the surface area of underlying bone in a milder model of posttraumatic OA. Combinatorial PRG4 and IL-1Ra gene therapy better preserved articular cartilage compared to monotherapy in a severe disease model [36].

Whilst these results look promising in murine studies, there is currently no IL-1Ra trials in humans looking at treating CACP. There are isolated reports where the IL-1Ra Anakinra has been given to children with CACP and they showed only a partial response, before switching to Methotrexate [37].

### Chondrodysplasias

Chondrodysplasias are a heterogeneous group of developmental disorders in which there are anatomical abnormalities and frequently observed early onset OA. The anatomical abnormalities result from dysregulated chondrocyte function in growth plate cartilage whereas the early onset OA may partly result from aberrant mechanical loading, as a consequence of altered joint shape, but also because the factors involved in cartilage development probably play an important role in homeostasis of mature chondrocytes. These conditions are often rare and typically severe in the phenotype they produce. Many of the genetic mutations that cause them are implicated in general OA, meaning that their severe and often single genetic mutation has given help in identifying pathogenic mechanisms or causative genes in the more common forms. Mutations in the gene encoding growth differentiation factor-5 (GDF5), a member of the transforming growth factor beta (TGFβ) superfamily, have been identified in several acromesomelic chondrodysplasias, which are a genetically heterogeneous mix of skeletal conditions including Hunter-Thompson [MIM:201250] and Grebe [MIM:200700].

### Hunter-Thompson

This condition is autosomal recessive inherited and commences neonatally. It is characterised by length abnormalities in the upper limb that include short proximal and middle bones and multiple abnormalities in the carpal bones, similar presentation is seen in the lower limb, which is more severely affected and both limbs demonstrate more severe presentation distally than proximally [38]. Recent novel homozygous mutation in the BMPR1B gene has been described to cause Hunter-Thompson type, as well as those mutations originally described in GDF-5 [39, 40].

#### Grebe

As with Hunter-Thompson, Grebe is also autosomal recessive inherited and commences neonatally, such that severe shortening of the limb bones can be detected on obstetric ultrasound [41]. The lower limbs are more significantly impacted than the upper and the severity of impact proceeds along the proximal to distal gradient with the digits displaying most abnormalities; the fingers present with the appearance of short toes and the feet are typically valgus. There are often absence or fusion of carpal bones [38, 42].

The numbers of individuals who have these conditions are ultra-rare across the globe, although potentially underreported like many rare diseases, particularly in countries where screening tools are lacking [43]. It is unclear whether these affected individuals progress to have OA of any description. Although rare, the importance of the genetic mutations in GDF5 in these conditions and the phenotypic presentation show that GDF5 plays a pivotal role in the regulation of chondrogenesis during joint development and regulates proteoglycan synthesis in mature cartilage. Interestingly, GDF5 has also been identified as a gene with one of the highest associations with OA from the large-scale genetic studies on OA-susceptibility [44, 45]. Its presence has been detected in adult and mice articular cartilage and its regulation is increased in OA [46–48]. Intra-articular recombinant human GDF5 supplementation has been reported to stop the disease progression and stimulate cartilage repair in a rat medial meniscus transection model of OA. An intra-articular disease modifying OA therapy is a potential target for OA therapy [49].

Research on chondrodysplasias has also revealed the potential role of endoplasmic reticulum stress (ER stress) as a disease mechanism not only in the pathogenesis of rare cartilage syndromes but potentially in common OA [50]. ER stress occurs when the ability of the endoplasmic reticulum to fold proteins is defective, leading to impaired cell function and if severe enough, to cell death. ER stress has been identified as a contributing mechanism in several chondrodysplasias.

#### Pseudoachondroplasia

Pseudoachondroplasia (PSACH) (OMIM #177,170), is autosomal dominant and results from mutation in the cartilage oligomeric matrix protein (COMP) gene. It is a disease that is typically not discovered or diagnosed until age 2–3 yrs, unlike GDF5 caused conditions which commence their phenotypic presentation neonatally, individuals with PSACH are typically normal in terms of growth at first but begin to fall behind their non-PSACH peers at approximately age 2. Radiographic examination reveals short long bones and smaller epiphyses and irregular metaphyses [51]. Patients typically progress to early onset OA in all major joints [52]. This condition has been termed a “COMPopathy” and is caused by protein misfolding resulting from defective calcium binding capabilities [53]. The mutant protein cannot be exported from the cell and collects intracellularly where it exerts significant ER stress and causes early death of growth plate chondrocytes—resulting in the shortened stature [54]. Recently, Resveratrol has been shown to reduce COMPopathy in mice through the activation of autophagic clearance of mutant COMP [55]. Resveratrol has been shown to have multiple benefits in a variety of systems, it has paradoxically been shown to also negatively impact others [56].

#### Multiple Epiphyseal Dysplasia

Multiple epiphyseal dysplasia (MED) (OMIM 600,969) (Fairbanks disease) is genotypically heterogeneous which brings some phenotypic variation in presentation. At least 6 different genetic mutations have been attributed to causing MED; 5 of them autosomal dominant: collagen type IX α-1 (COL9A1), collagen type IX α-2 (COL9A2), collagen type IX α-3 (COL9A3), (COMP) and matrilin-3 (MATN3). A mutation in the sulphate transporter gene SLC26A2 causes autosomal recessive MED [57]. Autosomal dominant cases outnumber recessive 3:1 and two-thirds of the dominant cases are attributed to mutations in the COMP gene, a quarter to MATN3 and the remainder to COL9A [57]. It is worth noting that there are cases of MED in the literature that as yet have unattributed mutations, suggesting that there are other genetic mutations to be discovered, where these patients have no mutations in any of the genes already known to be associated with MED [58]. Those individuals with dominant form MED usually present in childhood, with recessive individuals typically presenting in later life [58, 59]. Where mutations in the COMP gene are similar in MED to PSACH and thereby bring about similar pathogenic mechanisms for limiting bone growth and producing phenotype, it appears that there are likely to be a combination of intra- and extra-cellular factors that contribute to the disease pathology and as such may represent additional therapeutic opportunities [60]. Due to the current heterogeneity of the condition there is no treatment other than analgesia and the need for surgery and joint maintenance when deterioration occurs [61, 62].

#### Metaphyseal Chondrodysplasia Type Schmid

Metaphyseal chondrodysplasia type Schmid (MCDS) (OMIM: 156,500) is characterised by short stature that develops in the early years, with the absence of clinical or radiological features up to the age of 2 [63]. It is the least severe and most common of the metaphyseal chondrodysplasias with approximate 3–6 cases/million [64]. Almost all cases are caused by heterozygous mutation in the Col10A1 gene and many are de novo [65]. Biallelic variants have been associated with a more severe phenotype, although there is no genotype phenotype correlation in the literature [66] The significance of Col10A1 in this condition and its importance for understanding joint homeostasis and development is supported by the study of transgenic mice with the dominant negative type X collagen mutation showing skeletal deformities similar to those seen in MCDS and other chondrodysplasias [67] Similar to the ER induced stress seen in MED, Col10 is not exported from the cell correctly, causing an increase in intracellular ER stress. In OA, some specific markers of ER stress are increased and correlate with disease severity [68]. Pharmacological inhibition of ER stress responses by repurposing an antiepileptic drug carbamazine is being trialled as a therapy for metaphyseal chondrodysplasia type Schmid [69]. In vitro studies on chondrocytes have suggested markers such as peptides from Col II synthesis and ER stress markers (GRP78, CHOP/GADD153 and Caspase12) are reduced with the exposure to amino acids such as taurine or the flavonoid baicalin that target the H_2_O_2_ induced pathway [70, 71]. GRP 78 is the master regulator of the unfolded protein response. The inhibition of the pathways that these proteins are involved in alleviates chondrodysplasia by preventing aberrant chondrocyte differentiation [72, 73]. Positive reduction in ER stress has been shown to occur in vitro when cells are treated with trimethylamine N-oxide as well as lesser accumulation of defective protein in cells [74, 75].

## Rare Forms of Crystal Deposition Diseases

### Milwaukee Shoulder Syndrome

In 1857, Robert Adam first described the pathological features of what would become known as Milwaukee Shoulder Syndrome (MSS) [76]. McCarty et al. [77] formally identified the condition after encountering four elderly female patients with similar symptoms of shoulder joint arthropathy, rotator cuff defects, and large joint effusions**.** MSS is a rare disease linked to BCP crystal associated joint destruction [78]. It affects the shoulder joint, commonly causing rotor cuff defects in the absence of injury (bilaterally in 60% of cases), and gives an increased risk of OA in other joints, especially the knee [79]. The disease is unique in the degree of collagenase activity detected, resulting in free floating collagen types I, II and III in the synovial fluid [80]. This collagenase activity causes the absorption of intra-articular tendons and ligaments. Crystals can be identified using alizarin red staining of synovial fluid.

### Chondrocalcinosis

Chondrocalcinosis is the deposition of CPPD crystals in fibrous or hyaline cartilage. Most forms do not have a genetic component and are found in up to 50% of the population, and the presence of these crystals is common in elderly populations [81].

#### Chondrocalcinosis 1

Chondrocalcinosis 1 (CCAL1) (OMIM #600,668) (8q24.12) is a CPPD deposition disease associated with early onset OA. It is inherited in an autosomal dominant mode. Doherty et al. [82] detailed five English families affected by early onset chondrocalcinosis, one of which suffered with childhood convulsions. Baldwin et al., described 6 generations of a family with early onset OA (between 25 and 40 years) affecting both male and female members equally, where the localised genetic fault was chromosome 8q [83]. The authors suggested that raised levels of PPi in the synovial fluid could suggest a metabolic defect of cartilage [82], though the exact gene affected was not identified at the time of publication of these reports. A recent study involving a Dutch family, identified a gain of function mutation in tumour necrosis factor receptor super family member 11B (TNFRSF11B) as the gene responsible for CCAL1 [84]. TNFRSF11B codes for osteoprotegerin (OPG), a glycoprotein that regulates bone density and acts in the RANKL pathway inhibiting osteoclastogenesis and bone reabsorption. It has been suggested that any mutation in the TNFRSF11B gene causes a gain of function in OPG based on it being able to inhibit osteoclastogenesis, this has been seen in animal models but it is not reproduced in affected individuals [84, 85]. Most recently overexpression of CCAL 1 has been shown to be overexpressed in OA chondrocytes and its high expression correlates with OA severity [86].

#### Chondrocalcinosis 2

Chondrocalcinosis 2 (CCAL2) (OMIM #118,600) is a rare type of CPPD disease with an autosomal dominant inheritance pattern, though de novo mutations have been identified. It is often diagnosed in early adulthood. CPPD crystal deposits may occur on the vertebrae, causing stiffness. It is caused by gain of function mutations in the ANKH gene at location 5p15.2. The ANKH gene codes for a transmembrane protein that is expressed in joints and other tissues, its absence causes chondrocalcinosis [87]. Its function affects intra and extracellular levels of PPi. Increased levels of PPi promote the formation of CPPD crystals. Research into CCAL2 and other conditions linked to the ANKH gene are challenging due to the differing phenotypic presentation seen in the mice model compared to humans [88].

### Mechanism of Joint Degradation in Crystal Arthropathies

Although individually diseases such as Milwaukee shoulder syndrome, CCAL1 and CCAL2 are defined as rare, research into these diseases has taught us about more common forms of crystal deposition associated with OA. In the treatment of CCAL1 it has been suggested that therapeutics to counteract the action of OPG could prevent progression of disease [84]. In addition, BCP crystals are found in vascular calcification [89], and the RANK/RANKL/OPG pathway has been implicated in vascular calcification [90]. In vitro studies have shown that ANKH expression is modulated by cytokines, such as IL-1β and TNFα, as they decrease the amount of extracellular PPi, whilst TGFβ increases extracellular PPi concentrations [91]. This suggests that targeting the ANKH proteins could also be of therapeutic benefit [92]. Therefore, further research into rheumatological crystal deposition diseases may prove of interest to other disciplines.

Treatments, such as colchicine and allopurinol, are used to lower blood uric acid and prevent the formation of MSU crystals in gout and this relieves both the symptoms and removes the cause of disease. These treatments however have significant and common side effects [93]. Crystal deposits are ideal OA therapeutic targets, as the majority of current OA treatments focus on symptomatic relief, but removing or preventing the crystal deposits at an early stage could prevent acute and chronic BCP or CPPD diseases. However, this requires an accurate diagnosis of the underlying OA cause, which to date has proved difficult.

Current diagnosis tools for crystal deposition diseases are looking microscopically for crystals [94] in synovial fluid taken by arthrocentesis, which is a painful and invasive procedure. More accurate diagnoses are with more expensive and less readily available equipment, such as SEM and atomic force microscopy. Non-invasive techniques, such as MRI, ultrasound and X-ray can identify BCP and CPPD deposits [95], but these can’t distinguish between different crystal types and pick up later stages of disease. This poses challenges for screening for the true underlying causes of OA. Raman Spectroscopy can differentiate between different forms of crystals [96], and, although still in its infancy for in vivo imaging in bones [97, 98], has been used to non-invasively assess bone health [94] and could have applications for determining synovial fluid composition in vivo [99].

### Alkaptonuria (AKU)

AKU is a rare autosomal recessive condition caused by deficiency of homogentisate 1,2-dioxygenase (HGD) [100]. This enzyme sits on the tyrosine catabolic pathway where it is responsible for converting homogentisic acid (HGA) into maleylacetoacetic acid [101].

#### Clinical Presentation

The earliest presenting symptoms of this condition are not musculoskeletal, but urinary and present from birth. The clearance of HGA in urine leads to oxidation of the HGA molecule to form a benzoquinone intermediary, this chemical change causes a darkening of the urine [102, 103]. Over time, even with urinary clearance of HGA there is systemic elevation of the molecule within the body fluids and tissues and this leads to ochronosis which is macroscopically visible [104–108]. This ochronosis shows a preferential affiliation with articular collagenous tissues, as well as areas such as the sclera and cartilage of the ears. It is the continued deposition of the polymerised HGA that changes both the biochemical and biomechanical properties of the articular cartilages in AKU patients [102, 109].

AKU patients undergo early onset, rapidly progressing osteoarthropathy in multiple joints [110] eventually resulting in the need for surgical replacement. The certainty of this clinical progression and the causative gene being singular in defectiveness gives a unique opportunity to understand more about the underlying pathological mechanisms and the wider implications.

#### Macro- & Microscopic Features

In AKU, macroscopic examination of joint tissues clearly reveal that the cartilage undergoes the most notable changes, with non-uniform black colouration of the tissues. It is only in recent years that the pathogenesis of change in the cartilage of AKU patients has been elucidated [109]. Microscopic analysis of various AKU joint samples have shown that ochronotic pigmentation commences in the calcified cartilage zone, associated with individual chondrocytes and the pigmentation of the pericellular matrix of chondrocyte lacunae. Dense pigmentation can be seen without staining, but staining with Schmorls reagent highlights the presence of smaller ochronotic deposits [111].

In these areas of pigmentation of the calcified zone, there was a lack of pigmentation in any of the overlying zones or any evidence of changes in the articular surface of the cartilage that would be indicative of advanced arthritic changes [109]. Similar observations of the earlier stages and progression of the ochronotic phenotype have been observed in the mouse model of AKU. These observations are seen as early as 15 weeks of age in mice in the pericellular matrix, as with the human condition [112, 113]. This extracellular pigmentation was observed in the absence of similar intracellular histological staining of the relevant resident chondrocyte. Further progression at weeks 40 and 65 showed ochronosis to be seen in many cellular compartments and then throughout the femoral and tibial articular cartilage zones.

Whilst this pigmentation progress is extensive, there are still areas in both human and murine sections that demonstrate a lack of pigmentation. The question that remains is what is pre-disposing certain regions surrounding certain chondrocytes to undergo ochronotic pigmentation? HGA is systemically elevated in AKU and if this was the sole factor in determining pigmentation then the pigmentation should progress at a rate which affects all areas equally and commences at the same time across regions of tissues of a joint. As it is clearly not the case there must be other factors that cause the “marker” of HGA/pigment deposition in these specific chondrons. This may suggest that the HGA is acting as a marker for arthritic change in the tissue. It is clear that by end stage of the disease the presence of HGA polymer within the AKU chondrocytes indicates that cells are dead and not contributing to the maintenance, or the destruction of the surrounding matrix. In OA the chondrocytes contribute through multiple avenues to maintain the cartilage matrix; tissue anabolism, catabolism as well as inflammatory pathways [114]

The significance of the pericellular matrix in AKU and associated joint disease progression pre-dates the research focused on this region in OA. In OA the pericellular matrix is now recognised as the filter between the extracellular matrix and the chondrocyte(s) within a chondron [115]. The significance of the pericellular matrix in osteoarthritis is an important protective factor against the condition, it is clear that further research on the role of this area is needed [116].

The balance between anabolism and catabolism is a fine one, and the deviation from this results in OA disease. In AKU, the extracellular matrix is protected from the catabolic actions of the chondrocytes. This is due to the presence of the ochronotic pigment that is deposited in the extracellular matrix preventing any MMP’s degrading the matrix, it may also be that in the advanced stage of the disease the chondrocytes are so full of intracellular pigmentation that they are dead and then unable to produce MMP’s [110]. The extracellular matrix components of AKU cartilage are significantly changed compared to normal and OA cartilage. There is a significant reduction in extractable glycosaminoglycan’s (GAGs), as a result of the water soluble HGA in the cartilage matrix undergoing polymerisation in the tissue and binding it all in, or that it is lost so early in the disease process that it is no longer extractable. In either circumstance, the inability of GAGs to function in the cartilage milieu has a detrimental effect on the biochemical and biomechanical function of the cartilage [110]. It may be that this area in AKU tissues warrants closer scrutiny, the earlier onset and rapid progression of AKU may give an earlier time point to examine any changes than is seen in OA.

##### Osteoarthritis in Alkaptonuria

Genetic loss of homogentisate 1,2 dioxygenase in the liver results in the inability to break down homogentisic acid (HGA), which leaks into the circulation and over time binds to collagen in connective tissues, a process described as ochronosis. Cartilage is a favoured site for ochronosis, leading to severe osteoarthropathy. Nitisinone blocks tyrosine catabolism further upstream thereby preventing ochronosis.

In addition to focal loss and focal sclerosis of the subchondral plate in ochronotic joints, there is also aberrant remodelling of the underlying trabecular bone, which leads to further structural changes including the formation of trabecular excrescences; 1 of 3 types of new bone phenotypes seen in AKU surgical waste tissue samples. These novel microanatomical structures that were first identified in AKU where they are abundant in all samples but have been found subsequently in 70% of OA samples examined [117]. Interestingly adipocytes seem to play a role in the formation of trabecular excrescences either by direct contribution to synthesis or by templating the formation of bone by osteoblasts. AKU samples also showed a significantly altered bone and cartilage interface at the subchondral junction, with the complete erosion of articular calcified cartilage beneath a significantly biomechanical and biochemically altered hyaline cartilage overlying it [109].

The changes in the subchondral interface in AKU are extreme but not unique, there are significant changes in this region in OA with formation of microcracks that contribute to vascularisation, nerve growth and increases in the calcification of the deep cartilage zones, all of which play a role in the OA progression and symptomatic presentation [118, 119].

The progression of AKU into the cartilage of synovial joints causes early onset osteoarthritis, which may be identified radiographically. One method for monitoring progression of AKU is via macroscopic observation of ear cartilage, and histologically from biopsies. An alternative method for exploring the changes to cartilage is Raman spectroscopy, which is a laser-based technique that detects the inelastically scattered photons following interaction with a given tissue. Spectra provide the overall chemical measurement of a given sample, based on the energy exchange between the incident laser and energy used when the molecular bonds in a sample vibrate. In a study comparing non-ochronotic and ochronotic cartilage from hips and knees two main differences were found [120]. Specifically, non-ochronotic cartilage resulted in spectra comparable with that in the cartilage literature [121, 122], whereas the pigmented cartilage was highly fluorescent. The detectors on Raman instruments measure both Raman and fluorescence simultaneously; the former is chemically specific, whilst the latter is not. At a given wavelengths comparisons between these two outcome measurements may provide additional information, than Raman alone. In the AKU samples the fluorescence to Raman ratio correlated to the degree of pigmentation, i.e., the more pigmented a sample was the more it fluoresced and the less Raman detected. After removal of fluorescence from the remaining Raman spectral data, supported the exploration of the chemical progression of disease and the detection of spectral peaks that correlated with a spectrum of pure HGA [120]. Raman spectroscopy is a well-established laboratory technique, and due to its insensitivity to water and lack of ionising radiation it is being developed for clinical use, with several potential applications including osteoarthritis, other bone pathologies and cancer.

#### From Bench to Bedside

**Fig. 1:**
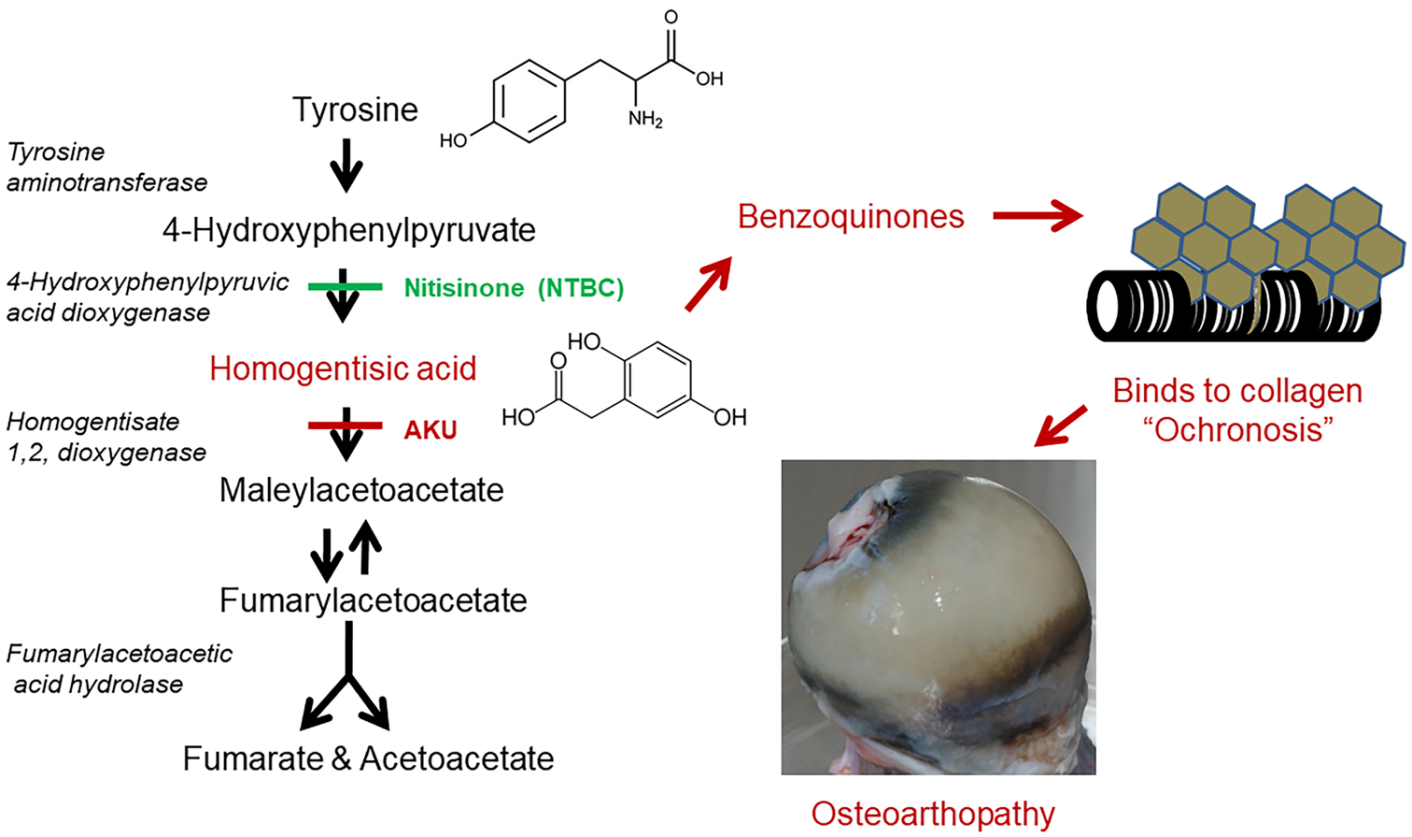
Tyrosine metabolic pathway and defect in AKU with theorised pathway of pigment binding to connective tissue matrix

AKU has benefitted from significant advances in recent years, with novel understanding of its pathogenesis and validation of in vitro and murine models leading to the approval of a treatment for this condition [108, 109, 111, 113, 123]. The administration of 10 mg Nitisinone daily to AKU patients has shown a 99.7% reduction in urinary HGA excretion, as well as a significant reduction in their disease scores compared to patients in the control group [124, 125]. This drug, which is routinely used for treatment of hereditary tyrosinaemia type 1 (HT-1) was also approved for use for patients with AKU by the European Medicines Agency on 22nd October 2020. The drug works by preventing the breakdown of the amino acid tyrosine by inhibiting the action of the enzyme 4-hydroxyphenylpyruvate dioxygenase. This acts to prevent the formation of tyrosine metabolites, such as HGA in AKU and fumarylacetoacetate, which converts to succinyl acetoacetate and succinylacetone which is highly mutagenic in HT-1 and leads to hepatocellular carcinoma (Fig. 1) [100].

Investigation of single samples of this rare, rapidly progressing OA, in the initial investigation of a 50 yr old male undergoing arthroplasty for lancinating pain in their hip, showed no loss of articular cartilage by macroscopic observation of the anatomy. Imaging by multiple techniques, MRI, microCT and scanning electron microscopy showed the presence of a hypermineralised substance protruding from the mineralising front of the calcified cartilage and extending into the soft, overlying hyaline cartilage [126]. These structures, termed high density mineralised protrusions (HDMPs) seemed to form through the microcracks in the subchondral plate allowing an initially fluid substance to penetrate before hardening in the spaces within the hyaline cartilage. The presence of these structures and the fact they are not full depth in their appearance in the hyaline cartilage raises questions about their function and formation. Whilst initially presumed to be disease specific to this rare form of OA, subsequent investigations have identified them in human OA joints and showing similarity to other structures seen in racehorses of varying breed and genetic origin [127–129]. Research in murine models of AKU has shown other novel structures, which as yet are still to be fully understood [130]

The discovery of these structures in rare human diseases such as AKU and then more commonly in human OA, as well as in other species suggests that there are still anatomical and pathological processes and structures to be discovered that are contributing to the mechanical and molecular forces that drive OA. Research into subchondral architecture and some of the conditions that potential OA genes associated with them have given a useful insight into how joint biomechanics may also contribute to OA initiation and progression—such as developmental dysplasia of the hip and femoroacetabular impingement [131–133]. Analysing these tissues and their associated structures with advanced imaging and targeted molecular techniques has the potential to drive future direction of pathogenic understanding as well as inform design and development of more specific and targeted therapeutic interventions to delay or reverse OA progression for millions of suffers.

## Conclusions

Greater focus on rarer forms of osteoarthritis has the potential to lead to more rapid advancement in understanding OA. Rare forms of arthritis are becoming useful tools in understanding the pathogenesis of more common forms as well as highlighting the significance of technological advances in being able to characterise subtle and significant differences in tissues. This is facilitated by the fact that many of these rare forms are the result of single gene defects. Individuals or groups with these rare disorders are already aware that the symptoms will at some point come to affect them and this is often at an earlier time point in life than those in the general population with more common aetiologies.
